# Supplementary material for: Identification and Evaluation of Diploid and Tetraploid Passiflora edulis Sims
Source: Plants (Basel). 2024 Sep 18;13(18):2603. doi: 10.3390/plants13182603 (PMC11434754; doi:10.3390/plants13182603)
Supplement: Supplementary file 1 [file plants-13-02603-s001.zip › plants-3202735-supplementary.pdf]

Supplemental information for

Identification and Evaluation of Diploid and Tetraploid *Passiflora edulis* Sims

Xin Su [1], Xue Wang [1], Ruilian Li [1], Chiyu Zhou [1], Lin Chen [2], Shi Chen [2], Nianhui Cai [1] and Yulan Xu [1,2,*]

[1] Key Laboratory of Forest Resources Conservation and Utilization in the Southwest Mountains of China, Ministry of Education, Southwest Forestry University, Kunming 650224, China; suxin@swfu.edu.cn (X.S.); wangwang199710@163.com (X.W.); fbfff@swfu.edu.cn (R.L.); zhouchiyu@swfu.edu.cn (C.Z.); cainianhui@swfu.edu.cn (N.C.)

[2] Key Laboratory of National Forestry and Grassland Administration on Biodiversity Conservation in Southwest China, Southwest Forestry University, Kunming 650224, China; linchen@swfu.edu.cn (L.C.); chenshi@swfu.edu.cn (S.C.)

* Correspondence: xuyulan@swfu.edu.cn

Supplementary Figure S1. PCA analysis on traits of diploid and tetraploid *P. edulis*

Supplementary Table S1. Stomatal parameters of diploid and tetraploid *P. edulis*

Supplementary Table S2. Growth index of diploid and tetraploid plants of *P. edulis*

Supplementary Table S3. Leaf parameters of diploid and tetraploid plants of *P. edulis*

Supplementary Table S4. Analysis of diurnal variation difference of net photosynthetic rate (μmol $m^{-2}\cdot s^{-1}$) between diploid and tetraploid *P. edulis*

Supplementary Table S5. Analysis of diurnal variation difference of transpiration rate (mmol m^{-2} s^{-1}) between diploid and tetra-ploid *P. edulis*

Supplementary Table S6. Analysis of diurnal variation difference of stomatal conductance (mol $m^{-2}s^{-1}$) between diploid and tet-raploid *P. edulis*

Supplementary Table S7. Analysis of diurnal variation of intercellular CO_2 concentration (μmol $m^{-2}s^{-1}$) between diploid and tet-raploid *P. edulis*

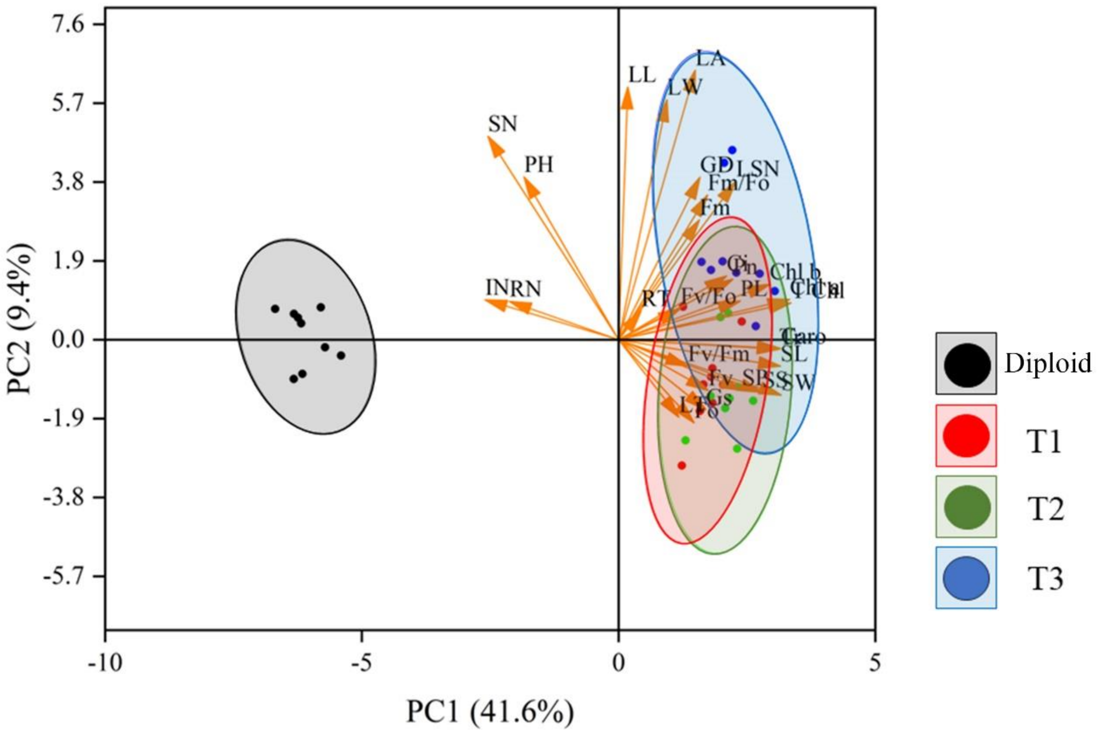

Supplementary Figure S1. PCA analysis on traits of diploid and tetraploid *P. edulis*. Diploid(black), tetra-ploid T1 (red), tetraploid T2 (green), tetraploid T3 (blue). PH: plant height; GD: ground diameter; IN: internode number; RL: root length; RN: root number; LT: leaf thickness; LL: leaf length; LW: leaf width; PL: petiole length; LA: leaf area; LSN: leaf serration number; SL: stomata length; SW: stomata width; SN: stomata number; SD: stomata density; Chl *a*: chlorophyll *a*; Chl *b*: chlorophyll *b*; T Chl: total chlorophyll content; Caro: carotenoid, SP: soluble protein; SS: solu-ble sugar.

Supplementary Table S1. Stomatal parameters of diploid and tetraploid *P. edulis*

Ploidy level	Line	Stomatal length (μm)	Stomatal width (μm)	Stomatal number	Stomatal density (No./mm^2)
2	Diploid	23.06±0.49b	18.35±0.39c	19.00±0.37a	2.81±0.05a
4	T1	33.16±0.53a	29.12±0.97b	8.78±0.22c	1.30±0.03c
4	T2	33.50±0.82a	32.32±0.56a	8.56±0.29c	1.27±0.04c
4	T3	33.50±1.02a	29.79±0.87b	15.56±0.29b	2.30±0.04b

Note: Different letters indicate significant differences, and the same letters mean insignificant differences ($P<0.05$). Duncan test.

Supplementary Table S2. Growth index of diploid and tetraploid plants of *P. edulis*

Ploidy level	Line	Plant height (cm)	Ground diameter (mm)	Internode number	Root length (cm)	Root number
2	Diploid	8.47±0.57a	1.27±0.10b	6.22±0.28a	4.66±1.35b	5.78±1.14a
4	T1	5.98±0.46b	1.42±0.06b	4.44±0.24b	7.77±2.08a	2.00±0.41b
4	T2	4.81±0.57b	1.52±0.10ab	3.89±0.31b	5.12±0.69ab	2.00±0.73b
4	T3	6.64±0.83b	1.75±0.10a	4.11±0.26b	5.48±0.97ab	2.11±0.56b

Note: Different letters indicate significant differences, and the same letters mean insignificant differences ($P<0.05$). Duncan test.

Supplementary Table S3. Leaf parameters of diploid and tetraploid plants of *P. edulis*

Ploidy level	Line	Leaf thickness (mm)	Leaf length (cm)	Leaf width (cm)	Petiole length (mm)	Leaf area (mm^2)	Leaf serration number	Leaf color
2	Diploid	0.10±0.01b	2.84±0.13a	1.39±0.08b	4.58±0.27b	416.32±2.03c	3.89±0.26d	Dark yellow-green
4	T1	0.13±0.00a	2.80±0.14a	1.61±0.06ab	7.33±0.31a	438.79±1.72b	5.11±0.26c	Dark green
4	T2	0.13±0.01a	2.80±0.20a	1.50±0.05ab	6.36±0.42a	417.03±1.42c	6.11±0.26b	Dark green
4	T3	0.12±0.01a	3.07±0.12a	1.85±0.23a	7.08±0.45a	577.06±2.63a	8.56±0.29a	Dark green

Note: Different letters indicate significant differences, and the same letters mean insignificant differences ($P<0.05$). Duncan test.

Supplementary Table S4. Analysis of diurnal variation difference of net photosynthetic rate (μmol m^{-2}·s^{-1}) between diploid and tetraploid *P. edulis*

Ploidy level	Line	8:00	10:00	12:00	14:00	16:00	18:00
2	Diploid	2.51±0.25b	5.40±0.12b	3.32±0.24b	1.96±0.11b	3.10±0.18c	2.68±0.11b
4	T1	4.37±0.70a	7.87±0.58a	5.90±0.43a	3.24±0.45a	5.19±0.25b	4.78±0.20a
4	T2	4.34±0.14a	7.61±0.53a	5.73±0.22a	3.56±0.20a	5.10±0.22b	4.58±0.30a
4	T3	4.09±0.23a	7.79±0.35a	5.32±0.45a	3.94±0.27a	5.87±0.23a	4.39±0.10a

Note: Different letters indicate significant differences, and the same letters mean insignificant differences (*P*<0.05). Duncan test.

Supplementary Table S5. Analysis of diurnal variation difference of transpiration rate (mmol m^{-2} s^{-1}) between diploid and tetra-ploid *P. edulis*

Ploidy levels	Line	8:00	10:00	12:00	14:00	16:00	18:00
2	Diploid	1.88±0.02b	2.34±0.07b	1.79±0.10b	1.56±0.12b	1.99±0.12b	1.70±0.10a
4	T1	2.82±0.07a	3.63±0.11a	2.42±0.12a	1.96±0.09a	2.97±0.15a	2.02±0.12a
4	T2	2.89±0.10a	3.64±0.10a	2.30±0.15a	2.10±0.08a	2.77±0.11a	1.96±0.14a
4	T3	2.97±0.15a	3.61±0.10a	2.42±0.12a	2.10±0.17a	2.72±0.12a	1.99±0.26a

Note: Different letters indicate significant differences, and the same letters mean insignificant differences (*P*<0.05). Duncan test.

Supplementary Table S6. Analysis of diurnal variation difference of stomatal conductance (mol m^{-2}s^{-1}) between diploid and tet-raploid *P. edulis*

Ploidy levels	Line	8:00	10:00	12:00	14:00	16:00	18:00
2	Diploid	0.36±0.01b	0.45±0.02b	0.31±0.02b	0.27±0.03a	0.41±0.01a	0.31±0.02b
4	T1	0.45±0.01a	0.51±0.00a	0.38±0.01a	0.36±0.01a	0.47±0.02a	0.40±0.01a
4	T2	0.45±0.02a	0.52±0.02a	0.38±0.01a	0.35±0.04a	0.46±0.03a	0.40±0.02a
4	T3	0.45±0.02a	0.51±0.02a	0.38±0.03a	0.36±0.04a	0.48±0.04a	0.40±0.02a

Note: Different letters indicate significant differences, and the same letters mean insignificant differences (*P*<0.05). Duncan test.

Supplementary Table S7. Analysis of diurnal variation of intercellular CO_2 concentration (μmol m^{-2}s^{-1}) between diploid and tet-raploid *P. edulis*

Ploidy levels	Line	8:00	10:00	12:00	14:00	16:00	18:00
2	Diploid	467.11±12.66b	460.22±6.40b	421.22±8.91b	280.11±2.75b	376.22±8.91b	426.22±6.40b
4	T1	560.44±26.18a	531.44±26.18a	459.56±0.99a	343.56±13.49a	402.56±0.99a	501.56±0.99a
4	T2	574.22±15.41a	545.22±15.41a	461.00±1.91a	330.78±5.22a	404.00±1.91a	503.00±1.91a
4	T3	568.00±14.23a	539.00±14.23a	468.89±3.72a	333.56±11.01a	411.89±3.72a	510.89±3.72a

Note: Different letters indicate significant differences, and the same letters mean insignificant differences (*P*<0.05). Duncan test.
